# Impact of SMTP Targeting Plasminogen and Soluble Epoxide Hydrolase on Thrombolysis, Inflammation, and Ischemic Stroke

**DOI:** 10.3390/ijms22020954

**Published:** 2021-01-19

**Authors:** Keiji Hasumi, Eriko Suzuki

**Affiliations:** 1 Department of Applied Biological Science, Tokyo University of Agriculture and Technology, Tokyo 183-8509, Japan; ersuzuki@cc.tuat.ac.jp; 2 Division of Research and Development, TMS Co., Ltd., Tokyo 183-0023, Japan

**Keywords:** SMTP, *Stachybotrys microspora*, triprenyl phenol, plasminogen, fibrinolysis, thrombolytic, soluble epoxide hydrolase, antioxidative, inflammation, stroke, cerebral infarction

## Abstract

*Stachybotrys microspora* triprenyl phenol (SMTP) is a large family of small molecules derived from the fungus *S. microspora*. SMTP acts as a zymogen modulator (specifically, plasminogen modulator) that alters plasminogen conformation to enhance its binding to fibrin and subsequent fibrinolysis. Certain SMTP congeners exert anti-inflammatory effects by targeting soluble epoxide hydrolase. SMTP congeners with both plasminogen modulation activity and anti-inflammatory activity ameliorate various aspects of ischemic stroke in rodents and primates. A remarkable feature of SMTP efficacy is the suppression of hemorrhagic transformation, which is exacerbated by conventional thrombolytic treatments. No drug with such properties has been developed yet, and SMTP would be the first to promote thrombolysis but suppress disease-associated bleeding. On the basis of these findings, one SMTP congener is under clinical study and development. This review summarizes the discovery, mechanism of action, pharmacological activities, and development of SMTP.

## 1. Introduction

The hemostatic system is finely regulated to achieve continuous blood circulation and prevent blood loss based on the balance between blood coagulation and fibrinolysis (blood clot degradation) [1,2,3]. The zymogens in this system contribute to autonomous regulation through spatiotemporal activation in response to physiological demands or stimuli [4,5]. Defects in the hemostatic system lead to bleeding or thrombotic disorders [6,7,8,9]. To date, several drugs have been developed to treat diseases related to or resulting from such defects in the hemostatic system. Particularly, antithrombotics, which inhibit blood clot formation, and thrombolytics, which accelerate the degradation of blood clots, are widely used to treat, control, or prevent thrombotic complications such as ischemic diseases of the heart, brain, lungs, and kidneys [10,11,12,13,14]. Despite several efforts to elaborate the use of these drugs, a significant population of treated patients suffer from bleeding events, which are occasionally severe or fatal [11,15,16,17]. Thus, it is believed that antithrombotics and thrombolytics are associated with an inherent inevitable bleeding risk [18].

In 1993, we initiated an investigation to identify a bioactive compound that controls the hemostatic system, particularly a small molecule that promotes endogenous fibrinolysis [4]. Our theory was that a molecule that modulates the physiological process will achieve a therapeutic effect without excessive bleeding risk. Using several systems to screen random microbial metabolites, we discovered multiple compounds with a novel activity, zymogen modulation [4]. *Stachybotrys microspora* triprenyl phenol (SMTP) is a class of identified compounds that modulate the conformation of plasminogen to promote plasminogen binding to fibrin or the cell surface [4,9]. SMTP is a large family of metabolites from *S. microspora*, comprising more than 60 congeners (Table S1) [19,20]. In agreement with our hypothesis, SMTP promotes thrombolysis without causing excessive bleeding [21,22,23,24]. Subsequently, we observed an unexpected additional function of SMTP, the inhibition of soluble epoxide hydrolase (sEH), which is a key enzyme that controls inflammation [25]. Along with the radical-scavenging activity inherent in the SMTP structure, the combination of thrombolytic and anti-inflammatory actions of SMTP played a key role in the treatment of ischemic stroke in several models of rodents and monkeys [21,22,24,26,27,28,29,30]. One of the SMTP congeners is under clinical development: compound development code, TMS-007; phase 1 study, JapicCTI-142654; and phase 2 study, JapicCTI-183842, registered at the Japan 540 Pharmaceutical Information Center Clinical Trials Information. Here, we review the discovery, mechanism of action, pharmacological activity, and development of SMTP.

## 2. Origin of SMTP

### 2.1. Background: Thrombotic and Thromboembolic Disorders and Treatment

Thrombotic and thromboembolic disorders are ischemic diseases that occur due to vascular occlusion by a blood clot formed in situ (thrombosis) or in an upstream vessel (embolism) that occludes a vessel at a downstream site [18,31,32]. The clot shuts off the supply of blood and oxygen, resulting in the death of the affected tissue. Thrombosis/thromboembolism can occur in both arteries and veins. Arterial thrombosis is the cause of most cases of heart attack (myocardial infarction) and ischemic stroke (brain infarction) [18]. Venous thrombosis/thromboembolism includes deep vein thrombosis, which accounts for most cases of pulmonary embolism (pulmonary infarction) [31]. These cardiovascular diseases constitute the most common causes of death in the developed world.

Atherosclerosis, or the rupture of an atherosclerotic plaque, is one of the most influential triggers for arterial thrombosis [31,33,34]. Atherosclerosis develops in the vessel wall through an accumulation of lipid deposits, mediated by macrophage foam cells that accumulate large amounts of cholesterol derived from lipoproteins such as low-density lipoprotein (LDL) that is oxidized in the vessel wall. Upon rupture of an atherosclerotic plaque, platelets rapidly aggregate to form a hemostatic plug through binding to collagen and von Willebrand factor. The aggregated platelets are activated to release several factors that promote the coagulation cascade and platelet aggregation/activation [18].

The coagulation cascade primarily consists of a sequential process of protease zymogen activation that results in the formation of fibrin and thrombus [2]. The exposure of coagulation factor VII to tissue factor, a transmembrane cell surface glycoprotein, is a pathophysiological trigger for the initiation of the coagulation cascade [2]. The hemostatic thrombus can be removed via another cascade reaction, the fibrinolytic system [35]. In both systems, the activation of protease zymogens is a key feature that regulates the local propagation of each event. The regulatory mechanism involves the instant response of the zymogen conformation to pathophysiological stimuli, triggering coagulation and fibrinolysis; the changes in conformation affect the localization and proteolytic activation of zymogens [4].

Generally, drugs targeting platelets (which inhibit platelet aggregation, resulting in the inhibition of blood clot formation) are used to treat arterial thrombosis [36], and venous thrombosis is treated with drugs targeting coagulation cascade proteases [37]. However, agents targeting the coagulation system are increasingly used in arterial disease, as evidenced by the COMPASS trial, where patients with stable atherosclerotic vascular disease were treated with a combination of Xa inhibitor and aspirin [38,39]. Although these drugs treat or prevent arterial and venous thrombosis/thromboembolism, a significant inherent risk of bleeding limits their use [18]. Another important class of drugs used to treat thrombotic/thromboembolic disorders is thrombolytics, such as tissue-type plasminogen activator (t-PA), which selectively cleaves plasminogen to form plasmin, a protease that degrades fibrin, the major component of blood clots [40]. However, the timing of thrombolytic intervention crucially affects the outcome: the earlier, the better. For example, t-PA therapy is beneficial only when used within 3 to 4.5 h of ischemic stroke onset, and the risk of intracranial hemorrhage increases significantly when used beyond this time window [41]. Thus, no drug that prevents blood coagulation or promotes thrombolysis without causing bleeding has been developed to date. Nevertheless, statins, a class of drugs that lower LDL cholesterol levels and suppress or retard atherosclerosis, are unique in that they reduce the risk of thromboembolic events such as heart attack and ischemic stroke without elevating the risk of bleeding [42]. However, statins do not directly treat thrombotic/thromboembolic diseases.

### 2.2. Search for a Bioactive Compound that Enhances Physiological Thrombolysis

One of the authors (K.H.) was involved in the identification of inhibitors of cholesterol biosynthesis and macrophage foam cell conversion led by Akira Endo, who discovered the first statin drug, ML-236B (compactin), and the second, monacolin K (lovastatin) [42]. Although we discovered several interesting molecules over more than 10 years of research, none of these compounds was developed further. By the early 1990s, several clinical trials had proven the clinical benefit of statin drugs for reducing LDL cholesterol, cardiovascular disease incidence, and mortality [42,43]. Meanwhile, we explored an approach to discover a new drug that directly controls thrombotic/thromboembolic disease through a hitherto unsought mechanism.

The theory behind our investigation was that a compound that enhances plasminogen binding to fibrin or the cell surface would promote physiological fibrinolysis, serving as an ideal approach to achieve regulated thrombus degradation. The basis of this theory was that (i) binding of plasminogen to fibrin or the cell surface is crucial for its activation to plasmin [44]; (ii) lipoprotein(a), a risk factor for cardiovascular diseases and atherosclerosis [45], competes with plasminogen for binding to fibrin and the cell surface [46]; and (iii) regulated fibrinolysis can occur without bleeding. Although this theory has no solid basis, especially regarding whether a small molecule could mediate protein (plasminogen)-to-protein (fibrin or receptor) binding, we started a pilot project to search for a molecule that enhances the binding of plasminogen to monocytoid cells (screening 1). Fortunately, a screening of random microbial cultures soon yielded several hits, including complestatin and its analog [47,48]. On the basis of these results, we expanded the project to screen for compounds that enhance plasminogen binding to fibrin (screening 2), cell-mediated fibrinolysis in plasma (screening 3), vascular endothelial cell surface generation of plasmin (screening 4), and reciprocal activation of plasminogen and single-chain urokinase-type plasminogen activator (scu-PA or prourokinase) (screening 5). These investigations led to the discovery of novel small molecules such as SMTP family, plactin family, and surfactin family compounds (see Table S1 and references therein). SMTP was discovered in screening 2. Notably, several compounds in these studies act through a unique mechanism, zymogen modulation [4].

### 2.3. Discovery of SMTP

Screening 2 identified several hits. Of these, an extract of a culture of *S. microspora* IFO 30018 (current repository code NCBI 30018) showed activity to enhance plasminogen–fibrin binding; however, isolation of the active principle was challenging, as the activity was distributed over a wide range of retention times with a turtle shell-like UV absorption peak, according to HPLC analysis. Initially, we believed that the mobile phase conditions were insufficient to achieve clear resolution; however, we subsequently realized that the broad turtle shell-like peak was due to several overlapping peaks with similar UV spectra. Repeated preparative HPLC fractionations corresponding to a relatively sharp convex shape in the turtle shell-like area yielded the first SMTP congener, staplabin (Table S1) [49]. The name staplabin was derived from *Stachybotrys* plasminogen-binding stimulator. Following the isolation of staplabin, we isolated two minor analogs, SMTP-1 and SMTP-2 (Table S1) [50]. Additionally, we developed a method to isolate multiple congeners by changing the composition of the culture medium to alter the turtle shell-shaped pattern to a truly peaked pattern. To clearly define each anticipated congener that would follow staplabin, we used a new designation, consisting of SMTP and a number.

### 2.4. SMTP Congeners

**Figure 1:**
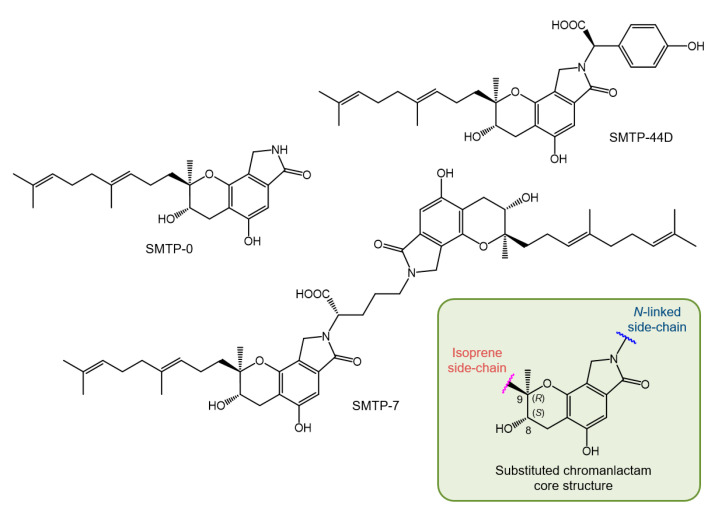
Structures of key *Stachybotrys microspora* triprenyl phenol (SMTP) congeners and substituted chromanlactam core unit.

The structure of the SMTP congeners consists of a chromanlactam moiety, an isoprene side chain, and a side chain linked to the nitrogen atom of the chromanlactam moiety (*N*-linked side chain) (Figure 1). Using the newly devised culture conditions mentioned above, we discovered six additional SMTP congeners, SMTP-3 to SMTP-8 (Table S1) [51,52]. These differed with respect to the *N*-linked side chains, all of which constituted known α-amino acids. Therefore, we hypothesized that the *N*-linked side chains originated from amino acids present inside the cell or in medium. To test this possibility, we used a poor medium in which amine compounds were restricted but which contained a specific amine to be incorporated as the *N*-linked side chain [53]. This precursor amine-feeding method enabled us to produce large amounts (up to 10 g L^−1^ of culture) of a specific SMTP congener quite selectively. This is a huge achievement, considering that the yield of staplabin was only 24 mg L^−1^ [49]. Furthermore, selective incorporation of the fed amine into the *N*-linked side chain was confirmed by the robust incorporation of rare amines such as D-amino acids following feeding [54]. Several SMTP congeners were isolated using a fermentation method that was fed with the precursor amine [19,20,25,55,56,57,58,59] (see Table S1 for details). Furthermore, some analogs differing in the isoprene side chain structure have been identified by microbial conversions of SMTP-0, which has a hydrogen atom as the *N*-linked side chain (Figure 1) [60].

### 2.5. Structure of SMTP

The structures of the SMTP congeners were elucidated using a combination of spectroscopic methods, including NMR and MS. The initial absolute stereochemistry was proposed utilizing NMR techniques using the simplest congener, SMTP-0 (Figure 1), and its derivatives [55]. The results obtained were consistent with the 8*S*, 9*S* configuration. However, a recent investigation that utilized a combination of NMR and crystallographic techniques proved an 8*S*, 9*R* configuration for an analog of SMTP, stachybotrin C (Table S1) [61,62]. Considering that stachybotrin C can be produced by feeding *S. microspora* with the precursor amine and that SMTP is produced from a common precursor, pre-SMTP (see Section 2.6), we conclude that all SMTP congeners exhibit an 8*S*, 9*R* configuration, and herein revise their stereochemistry (Figure 1).

### 2.6. Biosynthesis of SMTP

**Figure 2:**
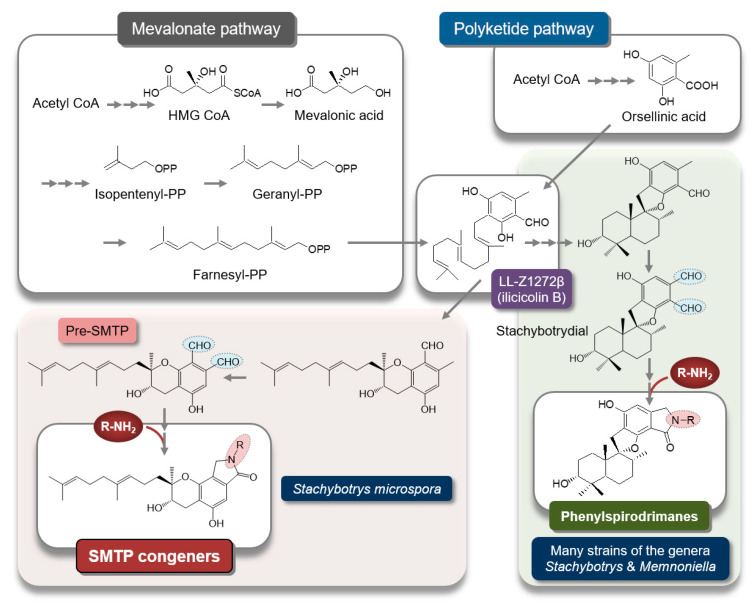
Putative pathway for SMTP biosynthesis. The synthetic pathway for SMTP-type and phenylspirodrimane-type triprenyl phenols may diverge, through differential cyclization mechanisms, from LL-Z1272β (ilicicolin B), a key intermediate synthesized from farnesyl diphosphate and orsellinic acid [63]. Pre-SMTP forms various SMTPs via a nonenzymatic reaction with an amine. Stachybotrydial [66] may yield a wide variety of phenylspirodrimanes via a similar mechanism, because aromatic *o*-dialdehydes (shaded in light blue) are highly reactive with an amine [67,68]. The precursor amine feeding method can be applied to selectively synthesize a phenylspirodrimane of interest [69] using *Stachybotrys* sp. F462, which forms stachybotramide, a phenylspirodrimane-type triprenyl phenol [70].

To elucidate the mechanism underlying the generation of a wide variety of SMTP congeners, we searched for a biosynthesis precursor of SMTP. We hypothesized that such a precursor might accumulate and disappear before and after amine feeding, respectively. We isolated two candidates: LL-Z1272β (ilicicolin B) and a novel compound. The latter, designated pre-SMTP, has no lactam but two aldehydes in the chroman moiety (Figure 2) [59]. Pre-SMTP spontaneously reacts with primary amines to yield an SMTP congener with the amine as an *N*-linked side chain. Thus, various SMTP congeners can be nonenzymatically derived from pre-SMTP. Incorporating relevant information from other studies [63,64,65], we propose an overall pathway for SMTP biosynthesis, as presented in Figure 2.

### 2.7. Other Triprenyl Phenols

The SMTP congeners belong to a large class of secondary metabolites, called triprenyl phenols, which are characteristic metabolites in the genera *Stachybotrys* and *Memnoniella*. Triprenyl phenols exhibit various biological activities depending on their structure [71], such as inhibition of the complement system [72], modulation of cholesteryl ester transfer among lipoproteins [70], antiviral and antiplasmodial activities [73], inhibition of avian myeloblastosis virus protease [74], inhibition of *myo*-inositol monophosphatase [75], inhibition of fucosyltransferases and syalyltransferases [76], inhibition of cholesterol esterase [77], endothelin-binding antagonism [78], inhibition of receptor tyrosine kinase [79], inhibition of glucose-6-phosphate translocase [80], suppression of lipid accumulation in hepatocytes [81], inhibition of HIV reverse transcriptase [82], antibacterial and antitumor activities [83,84], promotion of neurite outgrowth [85], inhibition of farnesyl-protein transferase [86], inhibition of squalene synthase [87], antiviral activity [88], and modulation of plasminogen conformation [4,89], described in detail in the following sections.

## 3. Biochemical Actions of SMTP

### 3.1. Enhancement of Plasminogen–Fibrin Binding and Plasminogen Activation

SMTP and staplabin (hereafter collectively referred to as SMTPs) were discovered through screening for a compound that enhances plasminogen binding to fibrin (see Section 2.2). Additionally, SMTPs promote plasminogen binding to cells. However, it was difficult to postulate that a small molecule could mediate a protein–protein interaction. A key to understanding the mechanism was obtained from the finding that SMTPs promote plasminogen activation to plasmin in the absence of fibrin or cells [90]. Thus, it was conclusive that SMTPs directly act on plasminogen.

**Figure 3:**
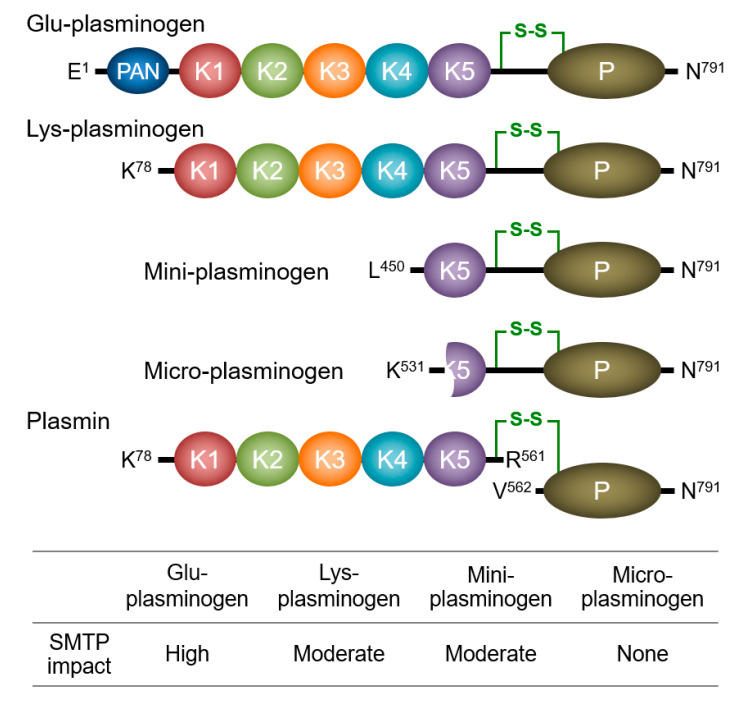
Plasminogen species. Schematic structures of the four plasminogen species and plasmin, and the effect of SMTP-7 on the activation of the four plasminogen species [97] are presented. A key disulfide bond connects the two chains of plasmin (green line). PAN, plasminogen–apple–nematode domain; K, kringle domain; P, protease domain.

**Figure 4:**
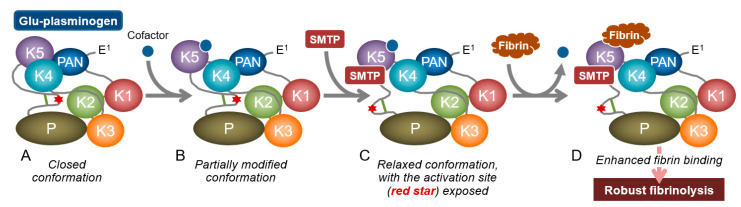
Proposed mechanism for the action of SMTP on plasminogen. (**A**) Schematic structure of Glu-plasminogen with closed spiral conformation, which is resistant to fibrin binding and plasminogen activator (PA)-catalyzed activation (see Figure 3 legend for designation of the symbol). The image incorporates essential structural information derived from crystallographic studies [94,95]. (**B**) An SMTP cofactor (phospholipid or unsaturated fatty acid) possibly binds to a site in K5 partly overlapping the aminohexyl site (see [98] for oleic acid–K5 binding) to modify the PAN–K5 interaction (image represents PAN–K5-disrupted molecule). (**C**) Cofactor-modified conformation may allow SMTP binding (possibly to K5, because SMTP does not affect micro-plasminogen, which lacks intact K5 [97]). SMTP binding leads to large-scale conformational change in plasminogen, enabling (**D**) robust fibrin binding and PA-catalyzed activation to plasmin (not shown). The resulting plasmin cleaves fibrin to yield C-terminal lysine, to which K1 (as well as K2 and K4) binds strongly, recruiting more plasminogen to accelerate fibrinolysis [99]. Thus, the promotion of plasminogen–fibrin binding and activation of the bound plasminogen in the early phase of fibrinolysis has a significant effect on the subsequent propagation phase. PAN, plasminogen–apple–nematode domain; K, kringle domain; P, protease domain.

To unveil the mechanism of SMTP action, it is essential to understand the conformational regulation of localized plasminogen activation. Circulating human plasminogen (Glu-plasminogen with Glu^1^) is a 791-amino acid single-chain glycoprotein consisting of, from the N-terminus, a plasminogen–apple–nematode (PAN) domain, five homologous kringle domains, and a serine protease domain (Figure 3) [35]. The cleavage at Arg^561^−Val^562^ by t-PA or u-PA forms an active two-chain enzyme, plasmin. Kringles 1, 2, 4, and 5 have a lysine-binding site. Kringle 5, unlike other kringles, can bind an internal lysine in addition to a C-terminal lysine. Thus, the kringle 5–lysine binding site is alternatively designated an aminohexyl site [91]. Native Glu-plasminogen adopts a tight conformation that is resistant to activation by plasminogen activators (PAs) (Figure 4). Once Glu-plasminogen binds to fibrin or cell surface receptors, or when it is converted to a truncated form, Lys-plasminogen (with Lys^77^ or Lys^78^ as an N-terminus; see Figure 3), it is readily activated to plasmin by PAs. Alternatively, treatment of Glu-plasminogen with a lysine analog such as tranexamic acid promotes PA-catalyzed activation (see the end of the paragraph for paradoxical antifibrinolytic action of lysine analogs). The common mechanism involved in these apparently different phenomena is the alteration of plasminogen conformation. The tight conformation is due to an intramolecular PAN (Lys^50^)–kringle 5 interaction [92,93]. Crystal structure analysis further reveals the involvement of Lys^50^, Arg^68^, and Arg^70^ of the PAN domain in the interaction with kringles 4 and 5 [94,95]. The binding of the kringle 5 aminohexyl site and an internal lysine in fibrin (located in D regions and αC-domains) leads to a conformational change in plasminogen, resulting in the formation of a fibrin–plasminogen–t-PA ternary complex that is crucial for the initiation of physiological fibrinolysis [96]. Lys-plasminogen lacks the PAN domain, and hence adopts an open conformation, which facilitates PA-catalyzed activation and fibrin binding. Lysine analogs compete with the lysine residue for the intramolecular PAN–kringle interactions, transforming the plasminogen conformation into an open conformation. Thus, the plasminogen conformation controls its localized activation. It should be noted that lysine analogs are used to block fibrinolysis, even these relax plasminogen conformation. This apparent discrepancy comes from the fact that lysine analogs compete with plasminogen for binding to fibrin (the plasminogen–fibrin binding is indeed mediated by the lysine-binding sites in plasminogen), emphasizing the importance of the plasminogen localization in fibrinolysis [4].

A model that depicts how SMTPs enhance plasminogen–fibrin binding and plasminogen activation was derived from the observations described in the next section.

### 3.2. Modulation of Plasminogen Conformation

The initial idea for the mechanism of action of the SMTPs [4,19,90] was based on the following observations: (i) SMTPs enhance the fibrin binding of both Glu-plasminogen and Lys-plasminogen; (ii) SMTPs enhance PA-catalyzed activation of both Glu-plasminogen and Lys-plasminogen, with higher enhancement for the former than the latter; (iii) SMTP-enhanced Glu-plasminogen activation is largely but not completely abolished in the presence of the lysine analog 6-aminohexanoic acid or a fibrin mimic; (iv) SMTP-enhanced Lys-plasminogen activation is not affected by the lysine analog or fibrin mimic; and (v) in size-exclusion HPLC, the elution of both Glu-plasminogen and Lys-plasminogen is advanced in the presence of SMTPs, representing an increase in the apparent molecular volume of plasminogens. All of these observations are consistent with a model in which SMTPs induce a conformational change in plasminogen [4]. The following additional results [97] enabled us to propose a refined model: (vi) the SMTP action depends on a certain type of surfactant included in the buffer; (vii) phospholipids can be physiologically relevant cofactors; (viii) the cofactor-dependent SMTP effects are obtained with plasminogen molecules containing kringle 5, such as Glu-plasminogen, Lys-plasminogen, and mini-plasminogen, but not with micro-plasminogen, which lacks kringle 5 (see Figure 3 for molecular structures of truncated plasminogen species); and (ix) SMTP-promoted plasmin autoproteolysis is observed in Lys-plasmin and mini-plasmin but not micro-plasmin. Thus, kringle 5 is crucial for the action of SMTPs. Among the plasminogen species, the impact of SMTPs on activation is most prominent in Glu-plasminogen, which adopts a tight spiral conformation (Figure 4), supporting the conformational modulation mechanism. Details of the proposed mechanism are shown in Figure 4.

### 3.3. Concept of Zymogen Modulation

In addition to SMTP, several other molecules identified in our studies (Table S2) act by affecting the conformation of zymogen to alter its activation, leading to a comprehensive view that these are zymogen modulators. For example, complestatin [47], thioplabins [100], and stachybotrydial [85] modulate plasminogen conformation [101]. The lipopeptides surfactins/iturins [102,103] and glucosyldiacylglycerol [104] modulate a reciprocal zymogen activation between plasminogen and single-chain u-PA (prourokinase) by altering the conformation of plasminogen (in the case of surfactins/iturins) or single-chain u-PA (in the case of glucosyldiacylglycerol). The cyclic pentapeptide plactins [105,106] affect prothrombin conformation to alter its assembly with factor Xa, factor XIII, and Ca^2+^ into the prothrombinase complex and the resulting prothrombin activation [107]. Additionally, plactins target a unique protease zymogen, plasma hyaluronan-binding protein (pro-PHBP, or pro-factor VII activating protease; pro-FSAP) to enhance its autocatalytic activation [108]. Details of the zymogen modulation concept are reviewed in [4].

### 3.4. Unexpected Anti-Inflammatory Action of SMTP

In accordance with its mechanism of action, SMTP promotes plasma clot clearance in a pulmonary embolism model [19,109]. Nevertheless, we sought additional pathophysiological conditions that might be affected by SMTP. This was because the plasminogen/plasmin system is crucial for pericellular proteolysis, which plays a role in tissue remodeling, wound healing, angiogenesis, embryogenesis, and tumor growth/metastasis [110,111,112]. SMTP-7 (Figure 1), one of the most active congeners, exhibited significant pharmacological activity in several models. This included the suppression/amelioration of (i) tumor angiogenesis and tumor growth (patents 2 and 4) [109], (ii) hepatitis (patent 5), (iii) nephritis (patent 6), (iv) metabolic disease (patent 13), (v) ulcerative colitis (patent 14) [25], (vi) Crohn’s disease (patent 14) [25], (vii) Guillain–Barré syndrome (patent 14) [25], and (viii) cancer cachexia (patent 15). Initially, it was unclear whether the efficacy was derived from something other than the plasminogen-modulating activity, as mentioned above. The development of an SMTP congener, SMTP-44D (Figure 1), which lacks plasminogen-modulation activity [57], clarified this problem. SMTP-44D, as well as SMTP-7, were active in all five models tested (models iv–viii), thus revealing an unexpected activity of SMTP [25]. Inflammation is a pathological basis in most of the abovementioned models.

### 3.5. Soluble Epoxide Hydrolase as an Anti-Inflammatory Target of SMTP

To elucidate the anti-inflammatory activity of SMTP, we sought to identify a target using another non-plasminogen modulator molecule, SMTP-50, which was immobilized to generate an affinity bead. Affinity purification using mouse liver homogenate, followed by a peptide mass fingerprinting analysis, identified sEH as a candidate.

In mammals, sEH exists as a homodimer of a two-domain polypeptide, with 555 amino acids in humans [113]. The C-terminal domain exhibits epoxide hydrolase activity (C-EH), through which it hydrolyzes anti-inflammatory epoxy fatty acids such as epoxyeicosatrienoic acid (EET) [114,115]. C-EH can be a therapeutic target of several disease states including inflammation, neurological disorders, and pain [116,117]. The N-terminal domain exhibits phosphatase activity (N-phos), through which it hydrolyzes lipophilic phosphomonoesters such as lysophosphatidic acids and sphingosine 1-phosphate [118,119]; however, no consensus has been reached on the physiological role of N-phos [120]. Deletion of sEH and inhibition of C-EH have similar consequences under multiple pathophysiological conditions, including insulin resistance [121], renal disease [122], psychiatric disorders [123], traumatic brain injury [124], atherosclerosis [125], ischemic stroke [126,127,128], hepatic steatosis [129], inflammatory bowel disease [130], and tissue/organ regeneration [131]. Therefore, it is postulated that most beneficial effects of sEH deficiency are ascribed to the loss of C-EH. Nevertheless, this theory does not negate the role of N-phos.

SMTPs inhibit both C-EH and N-phos activity [25]. C-EH inhibition by SMTP-0 is competitive, and N-phos inhibition is pseudo-noncompetitive. The C-EH-selective inhibitor 12-(3-adamantan-1-yl-ureido) dodecanoic acid (AUDA), which binds to the catalytic pocket, competes with SMTP-0 for binding. Thus, SMTP possibly binds to the C-EH catalytic pocket. The pseudo-noncompetitive mode of N-phos inhibition suggests an allosteric mechanism, whereas SMTP-0 binding to the C-EH pocket does not contribute to N-phos inhibition. Thus, SMTP-0 binds to two distinct sites in sEH.

SMTP can inhibit sEH both in vitro and in vivo, resulting in the decreased formation of dihydroxyeicosatrienoic acid (DHET), an sEH-catalyzed product from EET [25]. In a global analysis of 48 lipid mediators derived from the cyclooxygenase, lipoxygenase, or cytochrome P450 pathways in plasma from Guillain–Barré syndrome model rats, the plasma level of only one out of the 48 metabolites was significantly changed. This particular molecule was 11,12-DHET, which suggests that SMTP exerts a specific effect on the diverse metabolism of signaling lipids [25].

### 3.6. Antioxidative Action

The chroman moiety of SMTP is similar to that of tocopherols, which are potent antioxidants [132]. Therefore, several SMTP congeners exhibit potent antioxidative activity (patent 11) [57,133]. Notably, highly active SMTP congeners that have a carboxy- and/or hydroxy-substituted aromatic function in the *N*-linked side chain (see Section 3.7) are potent antioxidants. The antioxidative function contributes to the pharmacological activity of SMTP (see Section 4.2).

### 3.7. Structure–Activity Relationship

The assumption that SMTP binds to two independent sites on the sEH molecule to inhibit C-EH and N-phos implies that some SMTP congeners preferentially bind to either of the sites, resulting in varying selectivity in the inhibition of the two sEH activities, N-phos and C-EH. Indeed, SMTPs with different *N*-linked side chains exhibit varying inhibitory potency toward C-EH and N-phos [25]. Since the difference in the *N*-linked side chain affects the plasminogen modulation activity [56,57,58], we elucidated the comprehensive structure–activity relationship among plasminogen modulation, N-phos, and C-EH [20].

**Figure 5:**
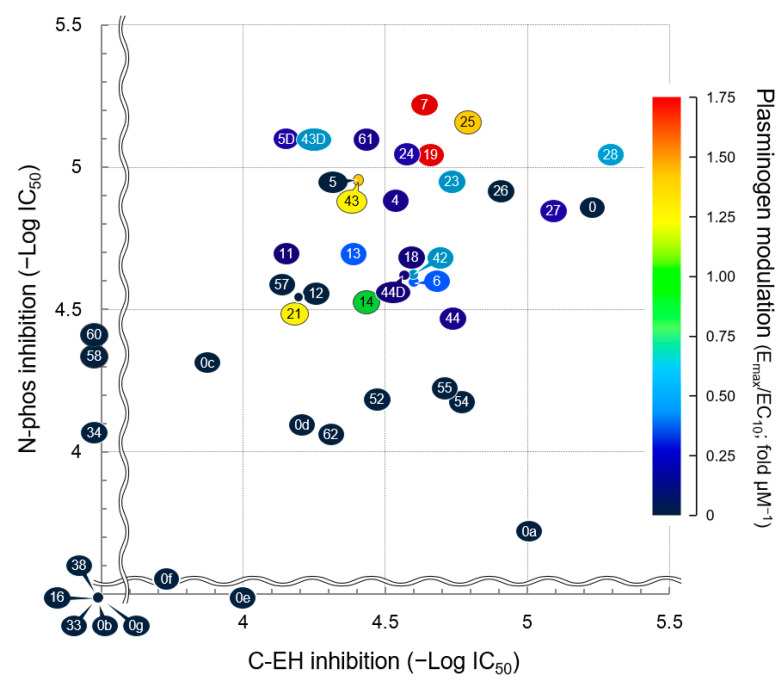
Structure–activity relationships of SMTP congeners with respect to plasminogen modulation and soluble epoxide hydrolase (sEH) inhibition. Plots are based on data in [20,60]. Plot number represents SMTP number (see Table S1 for structure). Details of the dimensions in the three axes are described in [20]. Briefly, the larger the value for each parameter, the stronger the activity (plasminogen modulation or sEH ihibition). C-EH, C-terminal epoxide hydrolase of soluble epoxide hydrolase; N-Phos, N-terminal phosphatase of soluble epoxide hydrolase.

As presented in Figure 5, the structure of the *N*-linked side chain of SMTP greatly affects its activity toward plasminogen modulation and N-phos/C-EH inhibition. For potent plasminogen modulation, both an aromatic group and a negatively ionizable group are essential as the *N*-linked side chain. Congeners with a carboxy- and/or hydroxy-substituted aromatic function are highly active, while congeners with a hydrophilic function or a highly hydrophobic function are essentially inactive. However, several congeners with a wide variety of *N*-linked side chains inhibit sEH. Notably, SMTP-0, a congener without an *N*-linked side chain, exhibits no plasminogen modulation capacity but potently inhibits both N-phos and C-EH equally. Thus, the core unit (chromanlactam with an isoprene side chain) is sufficient to exert N-phos/C-EH inhibition. Nevertheless, certain congeners with different *N*-linked side chains are considerably less active than SMTP-0, or they preferentially inhibit either N-phos or C-EH, demonstrating a role for the *N*-linked side chain in altering the potency and selectivity of sEH inhibition.

The structural requirement of the *N*-linked side chain for plasminogen modulation and for sEH inhibition are apparently different, as exemplified by SMTP-0. However, certain congeners with a carboxy- and/or hydroxy-substituted aromatic function in the *N*-linked side chain, such as SMTP-7, SMTP-19, SMTP-25, and SMTP-43, are highly active with respect to all three parameters. Since sEH-null mice, which lack both N-phos and C-EH, are resistant to various disease conditions that primarily involve inflammation [113], simultaneous inhibition of N-phos and C-EH can be an ideal means of pharmacological intervention in such diseases. In ischemic stroke, the combination of plasminogen modulation and sEH inhibition is essential for excellent pharmacological efficacy [29]. Therefore, a congener with all three activities can be a promising drug candidate for ischemic stroke. A congener without plasminogen modulation that inhibits sEH may serve as a potential candidate for targeting inflammatory diseases.

In addition to the *N*-linked side chain, the isoprene side chain affects sEH inhibitory activity. This conclusion was derived from experiments that evaluated a series of SMTP-0 derivatives isolated after microbial oxidative conversion of the isoprene side chain [60]. All of these derivatives exhibited low N-phos/C-EH inhibitory activity (Figure 5). In addition, analysis of the derivative SMTP-0a revealed an important independent role for the isoprene side chain. The C-EH inhibitory potential of SMTP-0a is approximately 50% that of SMTP-0, according to a cell-free enzyme assay, whereas the potency of SMTP-0a in a cellular system is only 1% that of SMTP-0. The fraction of cell-associated SMTP-0a is considerably lower than that of SMTP-0 [60]. Thus, the isoprene side chain contributes to the cellular localization of SMTP.

## 4. Pharmacological Activity of SMTP

While SMTPs have effectively ameliorated various pathological conditions in multiple animal models, as described in Section 3.4, this section focuses on their effect on ischemic stroke. Before describing the pharmacological activity of SMTP, we briefly review the epidemiology and etiology of ischemic stroke, as well as the current status of ischemic stroke treatment.

### 4.1. Ischemic Stroke and Treatment Strategy

Stroke is the second major cause of mortality and a leading cause of long-term disability worldwide [134,135]. One-third of stroke cases represent intracerebral or subarachnoid hemorrhage, while two-thirds are cerebral ischemia in 2013 across the world [135]. Ischemic stroke is commonly classified into the following three groups based on clinical manifestations: cardioembolic stroke, atherothrombotic stroke, or lacunar infarction [136]. Cardioembolic stroke, caused by cardiac embolus, accounts for 14–30% of ischemic stroke cases [137]. Generally, cardioembolic stroke is more severe, and results in a higher mortality rate than other ischemic stroke subtypes [138,139]. Atherothrombotic stroke is caused by an atherosclerotic plaque that narrows the lumen of the artery or occludes the vessel to induce blood clot formation; it accounts for 15–48% of ischemic stroke cases [140,141]. Infarction by atherothrombotic stroke often progresses in a stepwise fashion. Lacunar infarction is caused by the occlusion of a deep penetrating branch of 2013a cerebral artery by microatheroma and lipohyalinosis; it accounts for 15–34% of ischemic stroke cases [141,142,143]. Typically, the lacunar infarct area is small and the symptoms are relatively mild [144].

Ischemic stroke treatment is classified into two phases, acute phase treatment to rescue penumbra (viable but ischemic tissue) and chronic phase treatment to suppress secondary development of neuronal damage or to promote recovery from damage [145,146]. Recanalization therapy, especially thrombolysis and endovascular thrombectomy, is currently the major choice for acute phase treatment [147,148,149]. Among several thrombolytics developed to date, t-PA (alteplase) is highly recommended for ischemic stroke treatment (see [150,151,152] for historical and comparative perspectives on the thrombolytics). Despite the robust evidence backing t-PA therapy and endovascular thrombectomy, these therapies are not a panacea; they have some limitations. The use of t-PA is restricted to within 4.5 h (or 3 h) of stroke onset to avoid the deleterious effects of ischemia–reperfusion injury, such as fatal hemorrhagic transformation [153]. Thus, only 3–9% of patients with ischemic stroke receive t-PA therapy [147]. Endovascular thrombectomy is intended to treat large vessel occlusions [154]. It is estimated that less than 10% of patients with ischemic stroke qualify for thrombectomy [155], and few stroke centers have sufficient expertise in the procedure [156]. Thus, there is an urgent demand for a new therapy that overcomes these limitations, especially one that can benefit anyone (regardless of disease status) at any time (with a wide time window) and anywhere (regardless of resources/expertise).

### 4.2. Efficacy in Embolic Stroke Models

The first evidence suggesting the effectiveness of SMTP in ischemic stroke was obtained in experiments using a novel embolic stroke model in gerbils [26]. In this model, an acetic acid-induced thrombus that formed in the common carotid artery was released to embolize the middle cerebral artery. Following the success in the gerbil model, the same protocol was applied to mice [21,29,127,157]. Subsequently, SMTP was tested in another embolic stroke model in cynomolgus monkeys [28]. The observations from these experiments are summarized as follows: (i) SMTP-7 decreases infarct size, neurologic deficits, and edema [21,26,28]; (ii) SMTP-7 exhibits a much wider therapeutic time window than t-PA [21,26]; (iii) t-PA but not SMTP-7 causes intracranial hemorrhage [21]; SMTP-7 increases the level of plasmin–α_2_-antiplasmin complex in the plasma, which reflects plasmin formation in vivo [21,28]; (iv) SMTP-7 progressively restores blood flow, while t-PA causes rapid reperfusion [21]; and (v) SMTP-7 but not t-PA suppresses the expression of inflammatory cytokines in the infarcted brain tissue [21,157].

The structure–activity relationship study reveals some critical features of SMTPs, namely their thrombolytic, anti-inflammatory, and antioxidative activities [29]. A congener in which even one of these three activities is low has limited therapeutic potential in the mouse model of embolic stroke. Thus, the triad is essential for ischemic stroke treatment.

### 4.3. Efficacy in Thrombotic Stroke Models

SMTP-7 has been shown to be effective in thrombotic stroke models in mice [158] and cynomolgus monkeys [22]. In these models, an in situ thrombotic occlusion is induced by photoirradiation at the middle cerebral artery. In summary, SMTP-7 (i) decreases infarct size, neurologic deficits, and edema [22]; (ii) restores blood flow [22,158]; (iii) suppresses hemorrhagic transformation [22]; and (iv) suppresses the expression of inflammatory cytokines in the infarcted brain tissue [158].

### 4.4. Efficacy in Mechanical Cerebral Ischemia Models

In addition to the treatment of thrombotic/thromboembolic brain ischemia, SMTP-7 is effective in mechanically induced cerebral ischemia models such as transient and permanent focal ischemia [22]. Since these models do not involve thrombus, the efficacy might be attributable to the anti-inflammatory and/or antioxidative actions of SMTP-7. Indeed, several experiments with transient focal ischemia models demonstrated reduced levels of the following in SMTP-treated animals: (i) reactive oxygen species (ROS) [27,133]; (ii) markers of oxidative damage [24]; (iii) matrix metalloproteinase-9 (MMP-9) and blood–brain barrier (BBB) disruption [23,27]; (iv) inflammatory cytokines and neuroinflammation-related proteins [159]; and (v) hemorrhagic transformation [23,24]. Moreover, SMTP-44D, a non-thrombolytic but sEH-inhibitory congener, was shown to reduce oxidative damage markers and protect neurovascular units and trophic coupling from impairment in a mouse model of transient focal ischemia [30].

### 4.5. Effects on Bleeding and Hemorrhagic Transformation: Roles for Anti-Inflammatory/Antioxidative Actions

**Figure 6:**
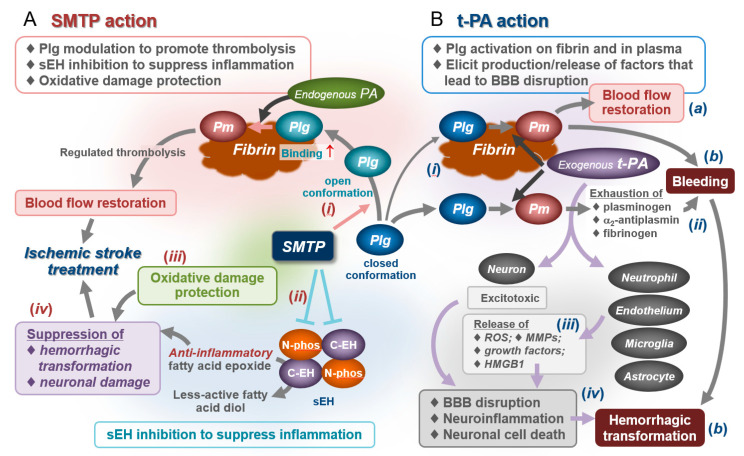
Summary of pharmacological activity of SMTP in comparison with tissue-type plasminogen activator (t-PA). (**A**) Pharmacological activity of SMTP is derived from a triad of activities: (*i*) modulation of plasminogen (*Plg*) conformation, promoting fibrin binding and conversion to plasmin (*Pm*), and subsequent fibrinolysis; (*ii*) inhibition of sEH, suppressing inflammation; and (*iii*) antioxidative activity, preventing oxidative damage. Process (*i*) is dependent on fibrin and endogenous PAs, allowing the SMTP action to promote physiological fibrinolysis. Therefore, pharmacological doses of SMTP do not cause bleeding. In process (*ii*), inhibition of C-EH-catalyzed hydrolysis of anti-inflammatory epoxy fatty acids such as epoxyeicosatrienoic acid (EET) may play a role; inhibition of N-phos may play an additional role (not shown). In process (*iii*), radical-scavenging activity inherent in the SMTP structure plays a role in the suppression of oxidative modifications of lipids, proteins, nucleic acids, and sugars [27,30,133,158,159], which are implicated in cytotoxicity, tissue damage, and inflammation. Anti-inflammatory/antioxidative effects contribute to the suppression of neuronal damage and hemorrhagic transformation (*iv*). SMTP’s triad of actions results in an excellent pharmacological effect for ischemic stroke. (**B**) Tissue-type plasminogen activator (t-PA) therapy is a powerful approach to treat ischemic stroke when administered within 4.5 h of onset (*a*); however, clinical outcome is poor when administered beyond this time window (*b*). t-PA therapy is associated with a significant increase in intracranial hemorrhage [153,163]. This can be explained by the following: (*i*) large amounts of pharmacological t-PA (exogenous t-PA) promote plasminogen activation in fibrin clots and in plasma; (*ii*) the latter induces the exhaustion of key hemostatic proteins such as plasminogen, α_2_-antiplasmin, and fibrinogen, resulting in increased bleeding risk; (*iii*) t-PA exhibits non-fibrinolytic action affecting neutrophils, vascular endothelial cells, microglia, and astrocytes to induce production/release of reactive oxygen species (ROS), matrix metalloproteinases (MMPs), growth factors, and high-mobility group box protein 1 (HMGB1), which cause neuronal damage, inflammation, vascular remodeling, and blood–brain barrier (BBB) disruption [163]; and (*iv*) the systemic bleeding tendency in combination with the non-fibrinolytic action of t-PA increases the risk of hemorrhagic transformation.

In normal mice and rats, SMTP-7 only slightly increases the level of plasmin–α_2_-antiplasmin complex, an index of plasmin formation in vivo [19,22], whereas the increase is prominent in embolic stroke models of mice and cynomolgus monkeys [21,28,29]. The difference arises most probably due to the mechanism of action of SMTP: modulation of plasminogen conformation to enhance plasminogen–fibrin binding and subsequent PA-catalyzed plasminogen activation in situ. There may be very few thrombi in normal physiological conditions, and the chance of fibrin/endogenous PA-dependent promotion of plasminogen activation by SMTP can be low under healthy conditions compared to pathological thrombosis conditions. The fibrin/endogenous PA-dependent mechanism may account for the difference in impact between SMTP and pharmacological t-PA on bleeding. t-PA but not SMTP-7 promotes bleeding at pharmacological doses in mice [22]. While pharmacological t-PA can drive plasminogen activation out of the range of the physiological process, as evidenced by the exhaustion of fibrinogen, plasminogen, and α_2_-antiplasmin [160,161,162], SMTP only promotes fibrin/endogenous PA-dependent fibrinolysis (Figure 6). Moreover, SMTP-7 does not affect the factors that potentially influence bleeding: blood coagulation, platelet activation, heart rate, blood pressure, rectal temperature, and blood physiological parameters [22].

Hemorrhagic transformation is a common complication of ischemic stroke [163]. The suppression of hemorrhagic transformation is a remarkable feature of SMTP. This is quite different from the property of t-PA, which increases the risk of bleeding and hemorrhagic transformation [153]. The abovementioned anti-inflammatory/antioxidative mechanisms can contribute to the suppression of ischemia–reperfusion injury, resulting in hemorrhagic transformation. The mechanism involved in the development of early hemorrhagic transformation (within 18–24 h after stroke onset) includes ROS and MMPs (leucocyte-derived MMP-9 and brain-derived MMP-2), which damage the neurovascular unit, resulting in disruption of the BBB. Delayed hemorrhagic transformation (>18–24 h) relates to ROS, MMP-2, MMP-3, MMP-9, and t-PA in the brain tissue, as well as neuroinflammation and vascular remodeling factors [163]. SMTPs reduce the levels of (i) inflammatory cytokines, (ii) MMP-9, (iii) ROS and markers of oxidative damage, (iv) neuroinflammation-related proteins, and (v) impairment of the neurovascular unit and trophic coupling; these observations support the theory that SMTP can actively reduce the risk of hemorrhagic transformation (Figure 6).

Thus, SMTP can serve as a drug that promotes recanalization (through enhancement of physiological fibrinolysis) while suppressing hemorrhagic transformation (through anti-inflammatory/antioxidative mechanisms) in ischemic stroke. The difference between SMTP and t-PA in this regard is schematically shown in Figure 6.

## 5. Development of SMTP

On the basis of the abovementioned chemical, biochemical, and pharmacological studies, a potent SMTP congener, TMS-007, is currently under clinical development. This section summarizes the course of the development.

### 5.1. Chemistry, Manufacturing, and Controls

TMS-007 for nonclinical and clinical studies is produced by fermentation using the precursor amine feeding method. Large-scale manufacturing using a fermenter can produce kilogram-order amounts of the TMS-007 drug substance. A formulation that enables the drug infusion has been developed.

In addition to the fermentation method, total synthesis of an SMTP congener has been attempted by certain groups [164,165]. Jacolot et al. eventually succeeded in total synthesis of stachybotrin C, an analog of SMTP [61]. However, the synthetic route consists of more than 10 steps, and the overall yield of stachybotrin C from 3,5-dihydroxybenzoic acid is roughly estimated to be less than 3%. The *N*-linked side chain of TMS-007 has additional chiral centers and is structurally more complex than that of stachybotrin C, suggesting an advantage of the fermentation method, which has a capacity of several grams per liter, over chemical synthesis production.

### 5.2. Nonclinical Studies

A drug substance manufactured in conformance with the guideline proposed by the International Council for Harmonisation of Technical Requirements for Pharmaceuticals for Human Use was used for nonclinical toxicological and safety assessment under the good laboratory practice guidelines. No serious issues that could hamper future clinical developments were observed in a series of examinations required for a first-in-human study of TMS-007.

### 5.3. Phase 1 Clinical Study

The first-in-human phase 1 clinical study of TMS-007 (registered as JapicCTI-142654 at the Japan Pharmaceutical Information Center Clinical Trials Information) was conducted at the University of Tokyo Hospital, Tokyo, Japan, from October 2014 to August 2015. The design was a randomized, placebo-controlled, double blind, dose-escalation, parallel group study consisting of five cohorts administered different doses (3, 15, 60, 180, or 360 mg per individual). The primary objective was to evaluate the safety and tolerability of a single intravenous dose of TMS-007, and the secondary objectives included the characterization of plasma pharmacokinetics and pharmacodynamics. Healthy men aged 20–45 years were enrolled and randomized 3:1 to receive an intravenous dose of TMS-007 or placebo.

The plasma TMS-007 level was observed to be a linear function of the dose. No symptom of bleeding was found. While safety assessments of t-PA in humans have shown exhaustion of fibrinogen and α_2_-antiplasmin [160,161,162], these hemostatic factors were not affected by TMS-007 treatment. The plasma level of plasmin–α_2_-antiplasmin complex slightly increased in some of the treated subjects, consistent with nonclinical observations in normal animals [19,22]. No serious adverse events were observed. Thus, TMS-007 exhibits favorable pharmacokinetic and safety profiles. Details of the phase 1 study will be published elsewhere.

### 5.4. Phase 2 Clinical Study

A randomized, placebo-controlled, double-blind, dose-escalation, parallel group phase 2 study of TMS-007 in patients with acute ischemic stroke was initiated in December 2017 (registered as JapicCTI-183842). This study was aimed at evaluating the safety and efficacy of a single-dose intravenous infusion of TMS-007 in symptomatic patients with ischemic stroke, including cardioembolic and atherothrombotic stroke and lacuna infarction, who were ineligible for t-PA therapy or endovascular thrombectomy. The dose of TMS-007 was set at 1, 3, and 6 mg kg^−1^. A total of 41 sites were involved in patient enrollment. A draft of the results of this study will be available in 2021.

## 6. Conclusions and Perspectives

More than 60 SMTP congeners have been identified. Several of them exhibit significant plasminogen modulation activity, and many exhibit inhibition of N-phos and/or C-EH of sEH. Of the three portions of the SMTP molecule, the *N*-linked side chain determines the potency and selectivity of action toward plasminogen and sEH. The isoprene side chain mainly contributes to cellular localization. The precursor amine feeding method enables us to selectively produce a large amount of an SMTP congener of interest, thus paving the way for clinical development. A novel concept, zymogen modulation to control its fate, has been derived from a series of explorative studies that led to the discovery of SMTP. SMTP relaxes the conformation of plasminogen to enable the protein to strongly bind to fibrin and promote physiological fibrinolysis without augmenting bleeding. The inhibitory activity of SMTP toward sEH can contribute to its anti-inflammatory activity in vivo. The triad of SMTP activities—plasminogen modulation, anti-inflammatory activity, and antioxidative capacity—is essential for its excellent efficacy in ischemic stroke treatment. A remarkable feature of SMTP efficacy is the suppression of hemorrhagic transformation, which is exacerbated by conventional thrombolytic treatments. No drug with such properties has been developed yet, and SMTP would be the first to promote thrombolysis but suppress disease-associated bleeding. The ongoing phase 2 study will reveal the therapeutic potential of SMTP in patients with ischemic stroke. Moreover, an SMTP congener with both thrombolytic and anti-inflammatory activities can be conceptually effective in the treatment of other thrombotic and thromboembolic diseases such as myocardial infarction and pulmonary embolism. An anti-inflammatory SMTP without thrombolytic power may serve as a drug candidate for the treatment of inflammatory diseases.

## 7. Patents

The following is the list of patents derived from the study of SMTP.

1. Hasumi, K.; Hu, W.; Ohyama, S.; Narasaki, R. Method for Selectively Producing Triprenyl Phenol Compound and Utilization of the Same Compound as Medicine. JP2000306840, filed 31 August 2000.
2. Hasumi, K.; Ohyama, S.; Harada, T.; Hu, W. Plasminogen Fragment Having Neovascularization-Inhibiting Activity and Method for Searching Compound Which Induce Production of the Plasminogen Fragment. JP2001328411, filed 19 September 2001.
3. Hasumi, K.; Hu, W. New Triprenylphenol Compound. JP2003015279, filed 23 January 2003.
4. Hasumi, K.; Hu, W. Medical Composition for Preventing and Treating Angiogenesis-associated Disease. JP2003015279, filed 23 January 2003.
5. Hasumi, K.; Maeda, F.; Mitsumori, K. Agent for Improvement of Hepatic Function. PCT/JP2006/302796, filed 17 February 2006.
6. Hasumi, K.; Yagasaki, K. Pharmaceutical Composition for Treatment or Prevention of Nephritis and Method for Producing Same. PCT/JP2006/318972, filed 25 September 2006.
7. Hasumi; K.; Nomura, Y.; Narasaki, R.; Tometsuka, C. Skin Aging Inhibitor, Cosmetic and Skin Care Preparation. JP2007036638, filed 16 February 2007.
8. Hasumi, K.; Kitano, Y.; Oish, H.; Koide, H.; Hasegawa, K.; Narasaki, R. Triprenyl Phenol Compound, Process for Production of Triprenyl Phenol Compound, and Thrombolysis Enhancer. PCT/JP2007/055749, filed 20 March 2007.
9. Hasumi, K.; Koide, H.; Narasaki, R. Triprenylphenol Compound and Thrombus Dissolution Accelerator. JP2008060249, filed 10 March 2008.
10. Hasumi, K.; Koide, H.; Narasaki, R. Triprenylphenol Compound and Thrombus Dissolution Accelerator. JP2008060250, filed 10 March 2008.
11. Hasumi, K.; Koide, H.; Hasegawa, K.; Nishimura, N. Antioxidant. JP2010019735, filed 29 January 2010.
12. Honda, K.; Hashimoto, T.; Shibata, K.; Hasegawa, K.; Hasumi, K. Cytoprotective Agent. PCT/JP2010/051711, filed 5 February 2010.
13. Hasumi, K.; Ishikawa, M.; Chikanishi, T.; Nishimura, N.; Hasegawa, K. Pharmaceutical Composition for Metabolic Syndrome, Obesity, Hyperglycemia, Hyperlipidemia and/or Fatty Liver. PCT/JP2010/053545, filed 4 April 2010.
14. Ishikawa, M.; Tanaka, I.; Shirafuji, T.; Hasumi, K. Prophylactic or Therapeutic Agent for Inflammatory Bowel Diseases or Autoimmune Peripheral Neuropathy. PCT/JP2011/058405, filed 1 April 2011.
15. Hasumi, K.; Suzuki, E.; Ogawa, N.; Otake, S.; Kitano, Y.; Hasegawa, K.; Nishimura; N. Soluble Epoxide Hydrolase Inhibitor. PCT/JP2012/054472, filed 23 February 2012.
16. Hasumi, K.; Suzuki, E.; Nishimura, Y.; Kitano, Y.; Hasegawa, K.; Nishimura; N.; Tsujihara, K. Chroman Derivative. PCT/JP2013/055729, filed 1 March 2013.

## Data Availability

The data presented in this study are available in this article and supplementary material.

## Supplementary Materials

The following are available online at https://www.mdpi.com/1422-0067/22/2/954/s1, Table S1: The SMTP congeners, Table S2: The search for bioactive molecules leading to the discovery of the concept of zymogen modulator.

## Abbreviations

AUDA	12-(3-adamantan-1-yl-ureido) dodecanoic acid
BBB	blood–brain barrier
DHET	dihydroxyeicosatrienoic acid
C-EH	epoxide hydrolase of the C-terminal domain of soluble epoxide hydrolase
EET	epoxyeicosatrienoic acid
LDL	low-density lipoprotein
MMP	matrix metalloproteinase
N-phos	phosphatase of the N-terminal domain of soluble epoxide hydrolase
PA	plasminogen activator
PAI-1	plasminogen activator inhibitor-1
PAN	Plasminogen–apple–nematode
ROS	reactive oxygen species
scu-PA	single-chain urokinase-type plasminogen activator
sEH	soluble epoxide hydrolase
t-PA	tissue-type plasminogen activator
u-PA	urokinase-type plasminogen activator
