# Supplementary material for: Impact of SMTP Targeting Plasminogen and Soluble Epoxide Hydrolase on Thrombolysis, Inflammation, and Ischemic Stroke
Source: Int J Mol Sci. 2021 Jan 19;22(2):954. doi: 10.3390/ijms22020954 (PMC7835936; doi:10.3390/ijms22020954)
Supplement: Supplementary file 1 [file ijms-22-00954-s001.pdf]

Supplementary materials for

Impact of SMTP targeting plasminogen and soluble epoxide hydrolase on thrombolysis, inflammation, and ischemic stroke

Keiji Hasumi [1,2,] * and Eriko Suzuki [1]

1 Department of Applied Biological Science, Tokyo University of Agriculture and Technology, Tokyo 183-8509, Japan; hasumi@cc.tuat.ac.jp (K.H.) and ersuzuki@cc.tuat.ac.jp (E.S.)
2 Division of Research and Development, TMS Co., Ltd., Tokyo 183-0023, Japan; hasumi@tms-japan.co.jp
* Correspondence: hasumi@cc.tuat.ac.jp; Tel.: +81-42-367-5710 (K.H.)

Table S1. The SMTP congeners.

Entry	Designation	Isoprene side chain	Core unit	*N*-linked side chain	Remarks	Ref.
1	Staplabin	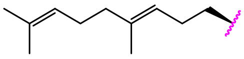 (Unit A)	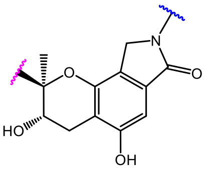 (Unit B)	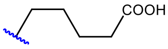	Prototype	[49]
2	SMTP-0	A	B	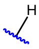	Simplest	[55]
3	SMTP-0a	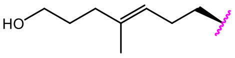	B	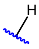		[60]
4	SMTP-0b	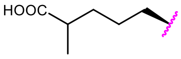	B	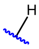		[60]
5	SMTP-0c	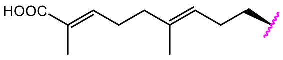	B	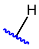		[60]
6	SMTP-0d	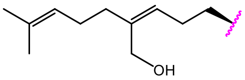	B	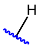		[60]
7	SMTP-0e	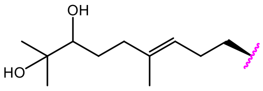	B	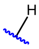		[60]
8	SMTP-0f	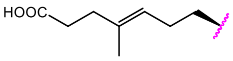	B	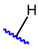		[60]
9	SMTP-0g	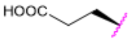	B	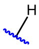		[60]
10	SMTP-1	A	B	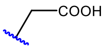		[50]
11	SMTP-2	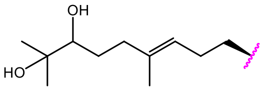	B	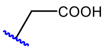		[50]
12	SMTP-3	A	B	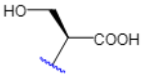		[51]
13	SMTP-4	A	B	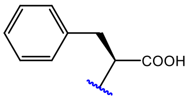		[51]
14	SMTP-4D	A	B	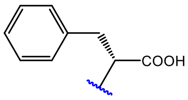		[54]
15	SMTP-5	A	B	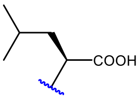		[51]
16	SMTP-5D	A	B	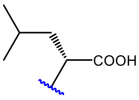		[54]
17	SMTP-6	A	B	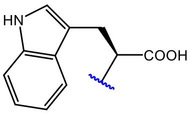		[51]

18	SMTP-6D	A	B	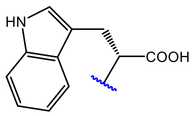		[54]
19	SMTP-7	A	B	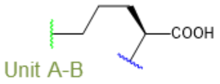	Intensively characterized	[52]
20	SMTP-7D	A	B	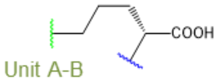		[54]
21	SMTP-8	A	B	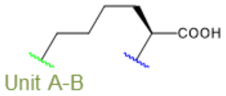	Potent	[52]
22	SMTP-8D	A	B	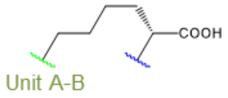		[54]
23	SMTP-9	A	B	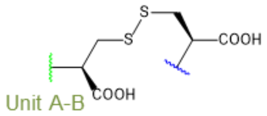		[19]
24	SMTP-10	A	B	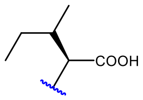		[56]
25	SMTP-11	A	B	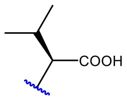		[56]
26	SMTP-12	A	B	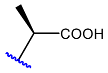		[56]
27	SMTP-13	A	B	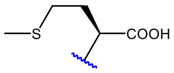		[56]
28	SMTP-14	A	B	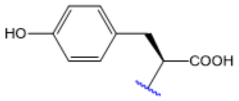		[56]
29	SMTP-15	A	B	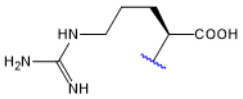		[56]
30	SMTP-16	A	B	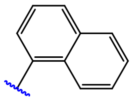		[56]
31	SMTP-18	A	B	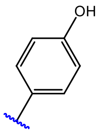		[57]
32	SMTP-19	A	B	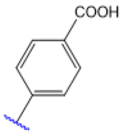	Potent	[57]
33	SMTP-20	A	B	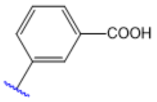		[57]

34	SMTP-21	A	B	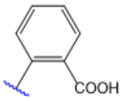		[57]
35	SMTP-22	A	B	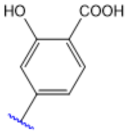	Potent	[57]
36	SMTP-23	A	B	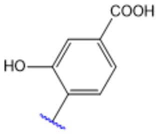		[57]
37	SMTP-24	A	B	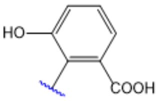		[57]
38	SMTP-25	A	B	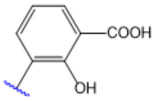	Potent	[57]
39	SMTP-26	A	B	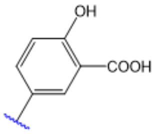		[57]
40	SMTP-27	A	B	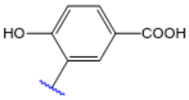		[57]
41	SMTP-28	A	B	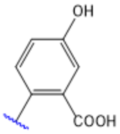		[57]
42	SMTP-30	A	B	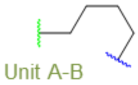		[19]
43	SMTP-31	A	B	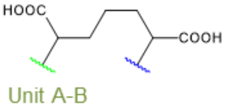		[19]
44	SMTP-33	A	B	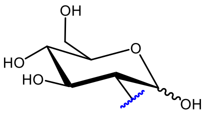		[56]
45	SMTP-38	A	B	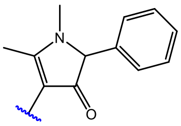		[56]
46	SMTP-40	A	B	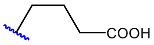		[56]
47	SMTP-42	A	B	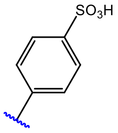		[56]
48	SMTP-43	A	B	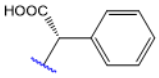	Potent	[58]

49	SMTP-43D	A	B	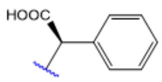		[58]
50	SMTP-44	A	B	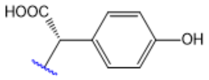		[58]
51	SMTP-44D	A	B	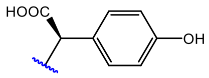	Well-characterized	[58]
52	SMTP-45D	A	B	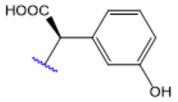		[58]
53	SMTP-46	A	B	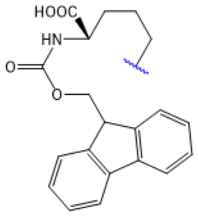	Fluorescent probe	Patent 9
54	SMTP-47	A	B	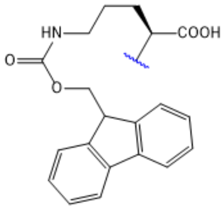	Fluorescent probe	Patent 9
55	SMTP-48	A	B	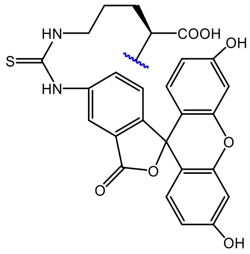	Fluorescent probe	Patent 10
56	SMTP-49	A	B	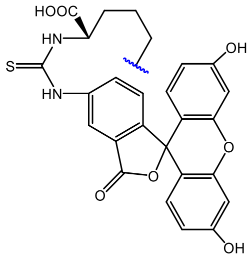	Fluorescent probe	Patent 10
57	SMTP-50	A	B	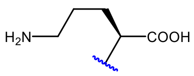	Affinity ligand	[25]
58	SMTP-52	A	B	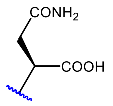		[20]
59	SMTP-54	A	B	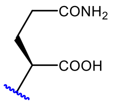		[59]
60	SMTP-55	A	B	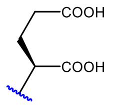		[59]

61	SMTP-57	A	B	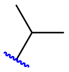		[20]
62	SMTP-58	A	B	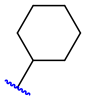		[20]
63	SMTP-60	A	B	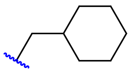		[20]
64	SMTP-61	A	B	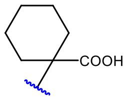		[20]
65	SMTP-62	A	B	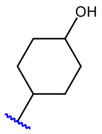		[20]
66	Pre-SMTP	A	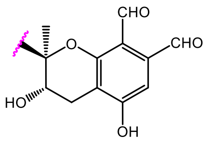	–	Biosynthesis precursor	[59]
67	Stachybotrin A	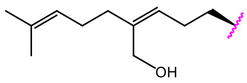	B (possibly)	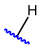	Possibly identical to SMTP-0d	[84]
68	Stachybotrin B	A	B (possibly)	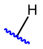	Possibly identical to SMTP-0	[84]
69	Stachybotrin C	A	B	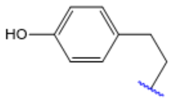	Identified as a neuritogenic	[85]

The following is the structure of unit A–B:

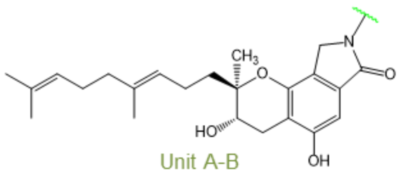

Table S2. The search for bioactive molecules leading to the discovery of the concept of zymogen modulator.

Screening	Target system	Compound identified	Mechanism
1	Plasminogen-cell binding	Complestatin [47]<br>Chloropeptin I [48]	Zymogen modulation (plasminogen) [101]
2	Plasminogen-fibrin binding	SMTP/staplabin<br>Thioplabin [100]<br>Stachybotrydial [89]	Zymogen modulation (plasminogen) [19,90,101]
3	Cell-mediated fibrinolysis in plasma	Plactins [105,106]	Zymogen modulation (prothrombin [107] and pro-PHBP [108])
		Malformins [S1,S2]	Altered plasminogen localization [S3,S4]
4	Vascular endothelial cell-surface generation of plasmin	Chaetoglobosin A [S5]	Induction of u-PA [S5]
		Crinipellin B [S5]	PAI-1 inactivation [S5]
		Geodin [S5]	PAI-1 inactivation [S5]
		Triticone B [S5]	PAI-1 inhibition [S5]
		11-Keto-9*(E)*,12*(E)*-octadecadienoic acid [S6]	PAI-1 inhibition [S7]
5	Reciprocal activation of plasminogen and scu-PA	Surfactins [102] and iturins [103]	Zymogen modulation (plasminogen) [102]
		Glucosyldiacylglycerol [104]	Zymogen modulation (scu-PA) [104]

PHBP, plasma hyaluronan-binding protein; PAI-1, plasminogen activator inhibitor-1; scu-PA, single-chain urokinase-type plasminogen activator.

References to Table S2

S1. Koizumi, Y.; Hasumi, K. Enhancement of fibrinolytic activity of U937 cells by malformin A_1. *J. Antibiot. (Tokyo).* **2002**, *55*, doi:10.7164/antibiotics.55.78.

S2. Koizumi, Y.; Nagai, K.; Hasumi, K.; Kuba, K.; Sugiyama, T. Structure–activity relationship of cyclic pentapeptide malformins as fibrinolysis enhancers. *Bioorganic Med. Chem. Lett.* **2016**, *26*, doi:10.1016/j.bmcl.2016.09.045.

S3 Koizumi, Y.; Fukudome, H.; Hasumi, K. Fibrinolytic activation promoted by the cyclopentapeptide malformin: Involvement of cytoskeletal reorganization. *Biol. Pharm. Bull.* **2011**, *34*, doi:10.1248/bpb.34.1426.

S4. Koizumi, Y.; Nagai, K.; Gao, L.; Koyota, S.; Yamaguchi, T.; Natsui, M.; Imai, Y.; Hasumi, K.; Sugiyama, T.; Kuba, K. Involvement of RSK1 activation in malformin-enhanced cellular fibrinolytic activity. *Sci. Rep.* **2018**, *8*, doi:10.1038/s41598-018-23745-0.

S5. Shinohara, C.; Chikanishi, T.; Nakashima, S.; Hashimoto, A.; Hamanaka, A.; Endo, A.; Hasumi, K. Enhancement of fibrinolytic activity of vascular endothelial cells by chaetoglobosin A, crinipellin B, geodin and triticone B. *J. Antibiot. (Tokyo).* **2000**, *53*, doi:10.7164/antibiotics.53.262.

S6. Shinohara, C.; Hasumi, K.; Chikanishi, T.; Kikuchi, T.; Endo, A. 11-Keto-9(*E*),12(*E*)-octadecadienoic acid, a novel fatty acid that enhances fibrinolytic activity of endothelial cells. *J. Antibiot. (Tokyo).* **1999**, *52*, doi:10.7164/antibiotics.52.171.

S7. Chikanishi, T.; Shinohara, C.; Kikuchi, T.; Endo, A.; Hasumi, K. Inhibition of plasminogen activator inhibitor-1 by 11-Keto-9(*E*),12(*E*)-octadecadienoic acid, a novel fatty acid produced by *Trichoderma* sp. *J. Antibiot. (Tokyo).* **1999**, *52*, doi:10.7164/antibiotics.52.797.
